# Design, Synthesis and *in Vivo* Evaluation of Novel Glycosylated Sulfonylureas as Antihyperglycemic Agents

**DOI:** 10.3390/molecules201119676

**Published:** 2015-11-06

**Authors:** Ghadeer A. R. Y. Suaifan, Mayadah B. Shehadeh, Rula M. Darwish, Hebah Al-Ijel, Vincenzo Abbate

**Affiliations:** 1 Department of Pharmaceutical Sciences, Faculty of Pharmacy, The University of Jordan, Amman 11942, Jordan; m.shehadeh@ju.edu.jo (M.B.S.); ghadeer_petra@yahoo.com (H.A.-I.); 2 Department of Pharmaceutics and Pharmaceutical Technology, Faculty of Pharmacy, The University of Jordan, Amman 11942, Jordan; rulamdarwish@yahoo.com; 3 Institute of Pharmaceutical Science, King’s College London, Franklin-Wilkins Building, 150 Stamford Street, London SE1 9NH, UK; vincenzo.abbate@kcl.ac.uk

**Keywords:** sulphonylurea, antihyperglycemic agents, glucosamine, streptozocine

## Abstract

Sulphonylurea compounds have versatile activities such as antidiabetic, diuretic, herbicide, oncolytic, antimalarial, antifungal and anticancer. The present study describes the design, synthesis and *in vivo* testing of novel glycosylated aryl sulfonylurea compounds as antihyperglycaemic agents in streptozocine-induced diabetic mice. The rational for the introduction of the glucosamine moiety is to enhance selective drug uptake by pancreatic β-cells in order to decrease the cardiotoxic side effect commonly associated with sulfonylurea agents. 2-Deoxy-2-(4-chlorophenylsulfonylurea)-d-glucopyranose was found to be the most potent antihyperglycaemic agents among the synthesized compounds in diabetic mice. This investigation indicates the importance of this novel class as potential antihyperglycaemic agents.

## 1. Introduction

Diabetes mellitus (DM) is a major degenerative disease with a serious cause of maladies in the 21st century [1]. The burden of diabetes is increasing globally, particularly in developing countries. In 2012, an estimated 1.5 million deaths were directly caused by diabetes [2] and in 2014, 347 million diabetic cases have been diagnosed worldwide. Moreover, Word Health Organization (WHO) estimated diabetes to be the 7th leading cause of death in 2030 [3].

DM is divided into three main types: Type I, Type II and Gestational diabetes. Type II diabetes mellitus (T2DM) accounts for more than 90% of all diabetic cases [4]. T2DM is a heterogeneous disease associated with both genetic and environmental causative factors including multiple defects in insulin secretion and action [5,6]. Insulin is a hormone that moves glucose inside the cells to produce energy. Upon inadequate insulin secretion, glucose level in the blood increases (hyperglycemia). Extended period of hyperglycemia causes irreversible damage to the eyes, kidneys, nerves and heart [7].

**Figure 1:**
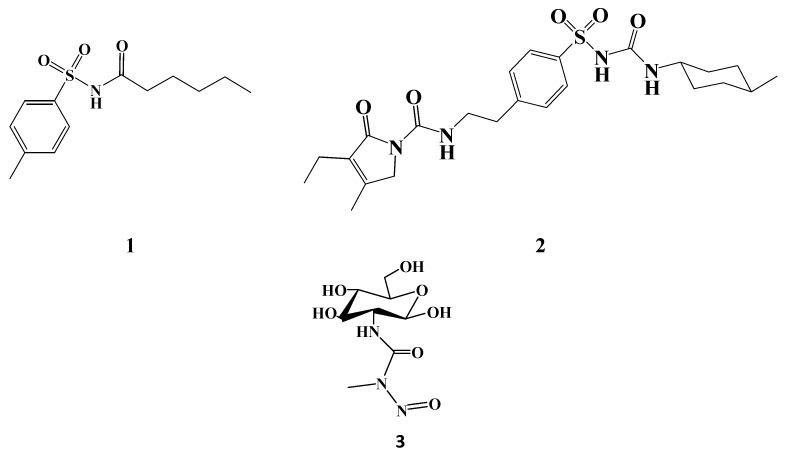
Chemical structures of Tolbutamide (**1**); Glimepiride (**2**) and Streptozotocin (**3**).

Hyperglycemia can be controlled by the administration of insulin which suppresses glucose production and augments glucose utilization. However, being ineffective upon oral administration, short shelf life, requirement of refrigeration, and in the event of over dose-fatal hypoglycemia limits insulin administration [8]. Despite extensive research efforts, only two classes of oral hypoglycemic agents (sulfonylureas and biguanides) are presently available as alternatives. Sulfonylurea agents act by increasing insulin release from β islet cells while biguanides act by reducing the excessive hepatic glucose production. Tolbutamide (**1**, Figure 1) was the first generation sulphonylurea drug developed and was later supplanted by the second generation (gliclazide, glipizide and glibenclamide (glyburide)) and the third-generation agent Glimepiride (**2**, Figure 1) [9]. However, these agents are associated with severe and sometimes fatal hypoglycemia, gastric disturbances like nausea, vomiting, heartburn, anorexia and increased appetite [10,11]. Nevertheless, these hypoglycemic agents are actively pursued since it is very difficult to maintain normoglycemia by any means in patients with DM. Therefore, the discovery of new hypoglycemic scaffolds with minimum side effects is still a challenge to medicinal chemists [12,13,14].

The clinical and medicinal importance of the arylsulfonyl urea moiety has been previously approved being an active pharmacophore, exhibiting various pharmacological activities. Literature review revealed sulfonylurea compounds with diverse biological activities such as antihyperglycaemia (e.g., glibenclamide) [15,16,17], diuretic (e.g., torasemide) [18], herbicide (e.g., chlosulfuron) [19,20,21,22], oncolytic (e.g., sulofenur) [23], antimalarial [24] antifungal [25] and anticancer [16,26,27]. Moreover, extensive research support the utilization of streptozotocin (STZ) (**3**, Figure 1), an atural chemotherapeutic agent isolated from *Streptomyces achromogenes* [28], as a selective toxin to pancreatic β-cells. Hence, it is utilized to create animal models of diabetes [29], or to treat pancreatic carcinoma [30]. The selective uptake and toxic effect of STZ against β-cells were previously unclear. However, recent studies have demonstrated that STZ is selectively transported by a glucose transporter (GLUT2) expressed in the pancreatic β-islet cells. This selective uptake is attributed to the presence of the glucosamine moiety acting as an analogue to *N*-acetylglucosamine, one of the cell wall petidoglycans [29,31,32].

In light of the above, we report the development of novel glycosylated sulfonylurea scaffolds by integrating the aryl sulfonamide with a glucosamine moiety to promote its selective uptake by pancreatic β-cells and to minimize adverse effects. The novel glycosylated sulfonylurea compounds were synthesized and evaluated for their hypoglycemic effect on normal (Group A) and diabetic (Group B) mice in comparison with the potent sulfonylurea antihyperglycaemic drug, Glimepiride (**2**).

## 2. Results and Discussion

### 2.1. Chemistry

Arylsulfonylurea derivatives were previously synthesized fromhazardous, irritant and moisture sensitive reagents such as arylsulfonyl isocyanates [33,34,35,36]. Later on, safer and more environmentally favorable methodologies have been developed to circumvent the use of these stoichiometric bases [37,38,39,40]. For example, tolbutamide (*N*-arylsulfonyl-*N*′-alkylureas) was synthesized by reacting benzenesulfonamides with *N*-alkylthiocarbonates obtained by selenium (or DMSO) assisted carbonylation of amines with carbon monoxide and sulfur [41,42]. Other methods used *N*,*N*-Dimethylaminopyridine (DMAP)-catalyzed reaction of amines with di-*tert*-butyldicarbonate[(Boc)_2_O] for the synthesis of isocyanates, which upon *in situ* trapping by an additional equivalent of amine, will produce unsymmetrical urea derivatives [21,43,44,45,46,47]. Alternatively, *N*,*N*′-unsymmetrical ureas were synthesized via the action of lithium methylpiperazine with the *N*-Boc-protected primary amines [48]. Other useful methods applied for the synthesis of ureas utilized cationic carbamoyl imidazolium salts which are derived from carbonyl diimidazole (CDI) [46]. However, it appears that (Boc)_2_O and CDI utilized in the above procedures were synthesized from phosgene. Accordingly, our targeted glycosylated sulfonylureas were synthesized according to the versatile, non-hazardous and practical synthesis of 4-dimethylaminopyridinium *N*-(arylsulfonyl) carbamoylide intermediates [38].

**Scheme 1:**
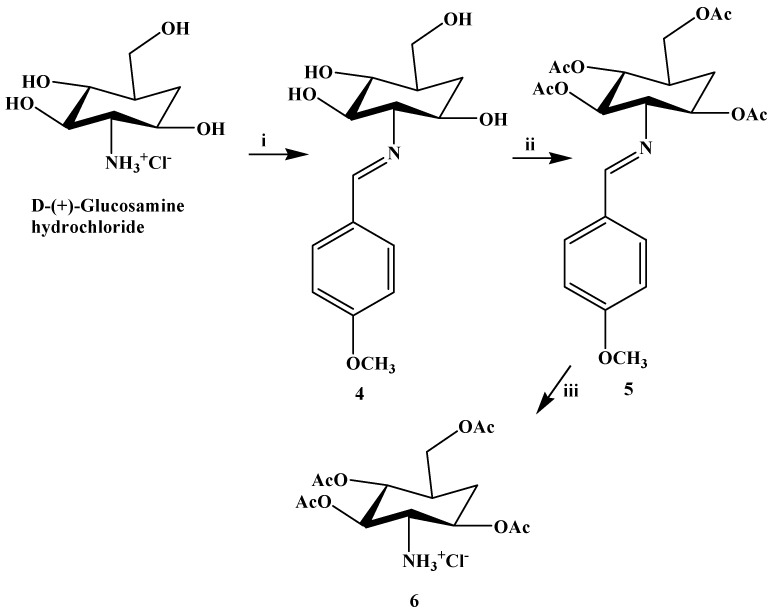
Chemical synthesis of compound **6**.

1,3,4,6-Tetra-*O*-acetyl-2-amino-2-deoxy-d-glucose hydrochloride (**6**, Scheme 1) was prepared from the commercially available d-glucosamine hydrochloride. Initially, the NH_2_ was protected by *p*-anisaldehyde (**4**) followed by acetylation (**5**) and removal of the *p*-methoxybenzylidene group with HCl in warm acetone (Scheme 1) [49].

(**i**) aq. NaOH, CH_3_O–C_6_H_4_–CHO; (**ii**) Py, Ac_2_O; (**iii**) acetone, 5 M HCl.

**Scheme 2:**
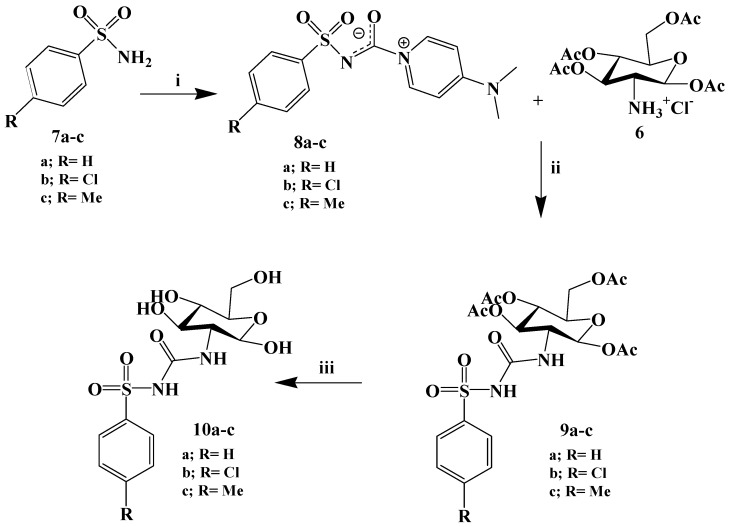
Chemical synthesis of compound **10a**–**c**.

On the other hand, the arylsulfonylcarbamoylides compounds were prepared as shown in Scheme 2. Sulfonamides (**7a**–**c**, 1 molar equiv.) reacted at room temperature with diphenyl carbonate (DPC, 1.1 molar equiv.) in the presence of DMAP (2 molar equiv.) in acetonitrile, to generate the title 4-dimethylaminopyridinium *N*-(arylsulfonyl)carbamoylides **8a**–**c** in 60%–68% yields. Neither prolonged reaction time nor elevated temperature changed the reaction course. Moreover, purification of the precipitated product was achieved by simple filtration from acetonitrile soluble by-products. Subsequent washing with diethylether removed excess DMAP. The above procedure worked well with phenylsulfonamide and its *para*-substituted congeners, such as *p*-methyl and *p*-chloro-phenylsulfonamide. The proposed mechanism of reaction for the preparation of compounds **8a**–**c** involves the initial replacement of DPC phenoxy group followed by formation of the pyridinium salt **8**. After this, a phenol molecule will be lost giving rise to the final product **8a**–**c**. Carbamoylide compounds were reported to be stable at room temperature for at least six months and indefinitely when refrigerated [21,38,50]. The stability of these highly polarizable adducts is mainly due to the delocalization of the positive charge on the pyridine ring and the negative charge on the arylsulfonylcarbamoyl moiety [21]. The chemical structure of compounds **8a**–**c** was confirmed by IR and ^1^H-NMR spectra. The IR spectra showed a vibration signal at 1694–1711 cm^−1^ for C=O. Moreover, the ^1^H-NMR spectra of compound **8a** showed the N–CH_3_ protons to resonate as a singlet at 3.21 ppm and a pair of doublets for the pyridinium ring protons appeared at 7.6 (3,5-CH) and 8.7 ppm (2,6-CH). Notably, these results were consistent with spectral data analysis of similar compounds [38].

(**i**) DPC, DMAP, MeCN; (**ii**) MeCN and Et_3_N; (**iii**) NaOMe, MeOH and Dowex 50WX8.

Direct coupling of d-glucosamine hydrochloride with carbamoylides **8a**–**c** under different reaction conditions turned out to be impossible. Thus, *O*-acetylated d-glucosamine hydrochloride was reacted with carbamoylides **8a**–**c** in acetonitrile at elevated temperature to afford the desired arylsulfonylureas **9a**–**c**. In a typical reaction, a slight excess of compound **6** (1.5 molar equiv.) was added in one portion to a solution of **8a**–**c** and Et_3_N (1.6 molar equiv.). The reaction mixture was refluxed for 5–30 min and then cooled to room temperature to afford sulfonylureas **9a**–**c** which were easily separated from the reaction mixtures following *in situ* acidification with 1% aqueous HCl. The identity of the newly synthesized arylsulfonylureas **9a**–**c** was proven by spectroscopic analysis. The infrared spectrum exhibited a characteristic absorption band at 1710 cm^−1^ in close resemblance to those for the carbamoylides **8a**–**c**.

The final *O*-deacetylated compounds **10a**–**c** were prepared according to the Zemplén procedure [51]. Following deacetylation, a mixture of products were always obtained, irrespective of whether the reaction was carried out by means of NaOMe in methanol, (CH_3_)_2_NH in methanol or K_2_CO_3_ in methanol-water solution [52]. The deprotection process was monitored by TLC using thymol/sulfuric acid as a detection reagent. Neutralization with Dowex 50WX8-200 ion-exchange resin afforded a crude mixture. Purification attempts by recrystalization using MeOH and acetone, afforded a crude gummy white solid. Thus compounds **10a**–**c** were purified by semi preparative HPLC. Notably, analysis of their ^1^H-NMR spectra drew our attention to the presence of α:β anomers. The thermodynamically more stable α-stereoisomer of glucosamine was predominated. The H-1 signal of the α-anomer appears at higher δ value compared to that of the β-anomer, owing to their different equatorial and axial orientations. This observation complies with the empirical rules of carbohydrates NMR spectroscopy [53].

### 2.2. In Vivo Evaluation

**Figure 2:**
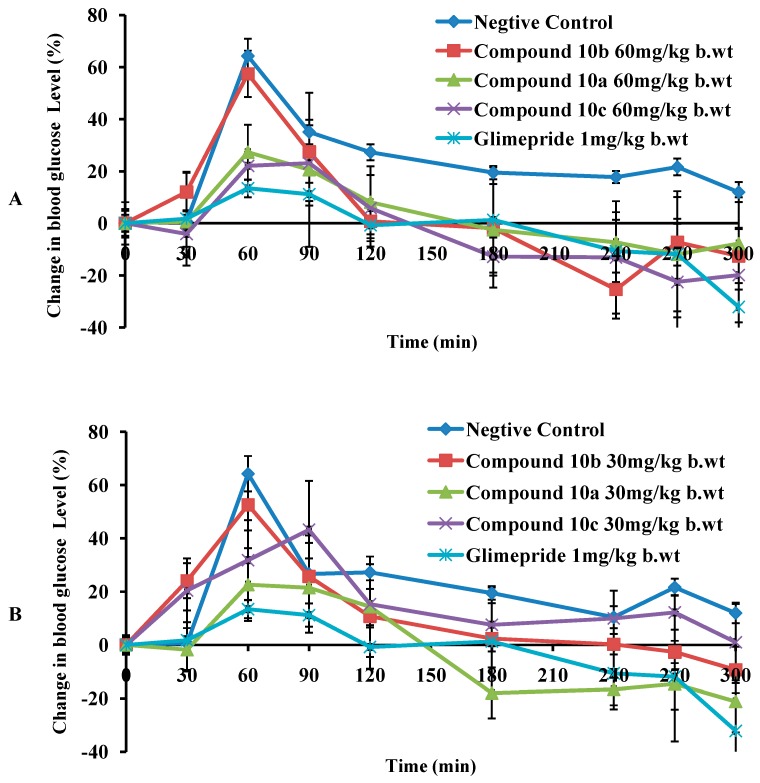
Change in blood sugar level in fasting normal mice. (**A**) **10a**–**c** (60 mg/kg b.wt); (**B**) **10a** and **10b** (30 mg/kg b.wt). Each bar results are the mean ± SEM for *n* = 5–8 rats per treatment group.

**Figure 3:**
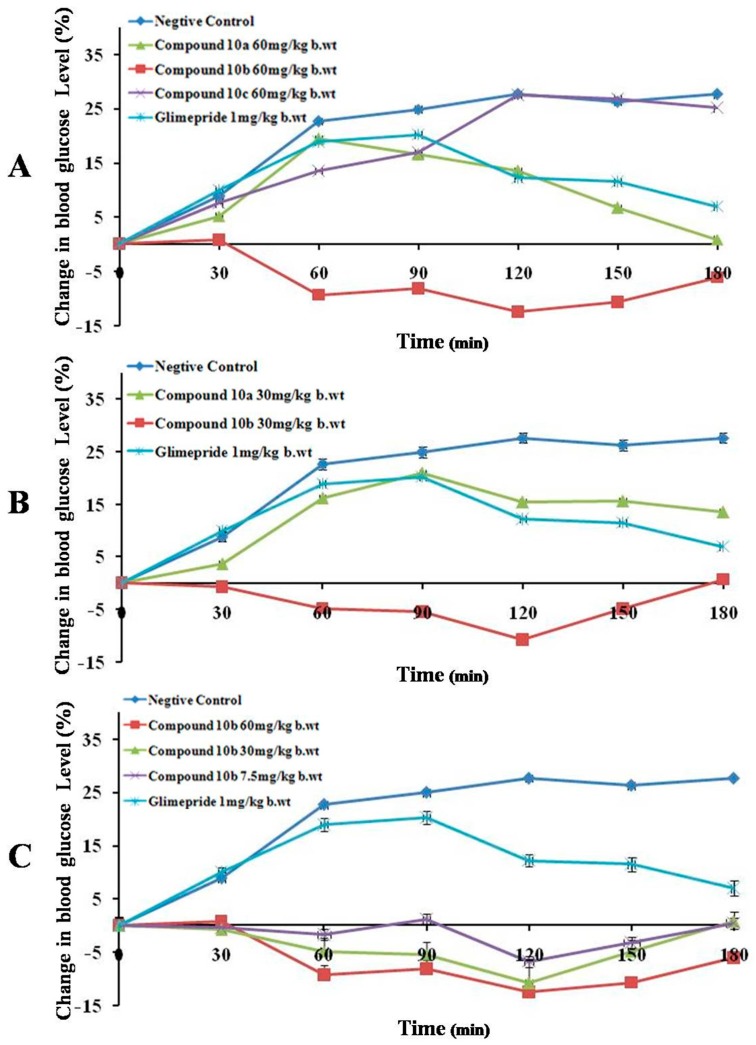
Change of blood sugar level in fasting STZ diabetic mice. (**A**) **10a**–**c** (60 mg/kg b.wt); (**B**) **10a** and **10b** (30 mg/kg b.wt); (**C**) **10b** (60 mg/kg, 30 mg/kg and 7.5 mg/kg b.wt). Each bar results are the mean ± SEM for *n* = 5–8 rats per treatment group.2.2.1. Normal Mice (Group A).

Administration of streptozotocin was previously reported to rapidly destroy pancreatic β-cells resulting in impairment of glucose-stimulated insulin release and induction of insulin resistance, both of which are associated with type II diabetes [54]. The antihyperglycaemic effect of different doses of compounds **10a**–**c** in normal (Group A) and STZ-induced (Group B) diabetic mice were assessed at different time intervals. The percentage change of glucose level from the initial fasting glycemia is shown in Figure 2 and Figure 3.

Figure 2A shows the change in blood glucose level in control and experimental normal mice received **10a**–**c** (60mg/kg body weight (b.wt.)) and Glimepiride. A raise up pattern to the highest blood glucose level was observed at 60-min time point of the test for the experimental, negative and positive control mice. The peak blood glucose level in the experimental and standard control mice was found to be lower than the negative control. In a comparable way, compound **10b** and the positive standard drug retained the blood glucose level to the fasting glycemia after 120 min. On the other hand, compounds **10a** and **10c** where less potent and took longer time. Tested compounds showed significant antihyperglycaemic effect over different time intervals when compared to the negative untreated control mice and standard Glimepiride as listed in Table 1.

**Table 1:** *P*-value for SPSS results in normal mice.

	Compound (Dose)	Time (min)								
		0	30	60	90	120	180	240	270	300
**A**	**10a** (60 mg/kg b.wt)	-	-	-	-	-	-	**	-	-
	**10a** (30 mg/kg b.wt)	-	-	-	-	-	*	*	*	**
	**10b** (60 mg/kg b.wt)	-	-	-	-	**	**	***	**	*
	**10b** (30 mg/kg b.wt)	-	-	-	-	**	**	**	*	*
	**10b** (7.5 mg/kg b.wt)	-	-	-	-	*	**		-	-
	**10c** (60 mg/kg b.wt)	-	-	-	-	-	*	**	*	-
	**10c** (30 mg/kg b.wt)	-	-	-	-	*	-	-	-	-
	Glimepiride 1 mg/kg b.wt	-	***	-	-	-	-	-	-	*
**B**	**10a** (60 mg/kg b.wt)	-	-	*	-	*	-	*	*	-
	**10a** (30 mg/kg b.wt)	-	-	*	-	-	***	*	-	**
	**10b** (60 mg/kg b.wt)	-	-	*	-	*	*	*	*	-
	**10b** (30 mg/kg b.wt)	-	-	-	-	*	**	*	*	-
	**10b** (7.5 mg/kg b.wt)	-	-	-	-	*	**	*	-	-
	**10c** (60 mg/kg b.wt)	-	-	-	-	-	**	-	-	-
	**10c** (30 mg/kg b.wt)	-	-	-	-	*	**	*	-	-

(**A**) * *p* < 0.05, ** *p* < 0.05 and *** *p* < 0.001 compared to control (untreated mice); (**B**): * *p* < 0.05, ** *p* < 0.05 and *** *p* < 0.001 compared to Glimepiride (Standard drug).

Figure 2B shows the change in blood glucose level in control and experimental mice received **10a**–**c** (30 mg/kg b.wt) and the Glimepiride. Decreasing the dose **10b** delayed the time requested to revert back to the fasting glycemia by an hour (*i.e.*, at 180 min-point). Notably, compound **10a** was able to maintain low blood glucose level. In general, compounds **10a**–**c** steadily exerted antihyperglycaemic effect at the tested doses when compared to the negative control.

#### Diabetic Mice (Group B)

Figure 3A shows the change in blood glucose level in control and experimental diabetic mice received **10a**–**c** (60 mg/kg b.wt.) and the standard drug. Glimepiride induced significant (*p* < 0.005) blood glucose reduction after 90 min. Compounds **10a** and **10b** at 60 mg/kg dose maintained lower blood glucose level compared to the untreated mice (negative control) at different test intervals. It was observed that the peak glycemia in the negative control mice rose from initial level to the peak value at 60–90 min time point and remained almost steady after 90 min. Whereas, Glimepiride produced a slight but significant (*p* ˂ 0.05) fall in blood glucose level as shown in Table 2. Maximal drop down in glucose level, below the initial glycemia, was observed in mice treated with **10** at 60 mg/kg and 30 mg/kg (Figure 3A,B). Remarkably, compound **10b** exhibited a significant antihyperglycaemic effect at 60 mg/kg, 30 mg/kg and 7.5 mg/kg over different time intervals when compared to the negative untreated control mice and standard Glimepiride as shown in Figure 3 and Table 2.

Compound **10a**, at 60 mg/kg, exhibited slower onset of hypoglycaemic action when compared to **10b** as the blood glucose level retrieved back to the fasting glycemia after 180 min. On the other hand, its antihyperglycemic effect was comparable with Glimepiride at different time intervals. Decreasing the dose of **10a** to half (30 mg/kg) lowered its antihyperglycemic effect as shown in Figure 3B. Compound **10c** illustrated a shorter duration of action with a noticeable diminish in its antihyperglycemic effect at 120 min time point.

**Table 2:** *P*-value for SPSS results in diabetic mice.

	Compound (Dose)	Time (min)						
		0	30	60	90	120	150	180
**A**	**10a** (60 mg/kg b.wt)	***	***	***	***	***	***	***
	**10a** (30 mg/kg b.wt)	-	-	-	-	-	-	-
	**10b**(60 mg/kg b.wt)	***	***	***	***	***	***	***
	**10b** (30 mg/kg b.wt)	-	*	***	***	***	***	***
	**10b** (7.5 mg/kg b.wt)	-	-	**	***	***	**	**
	**10c** (60 mg/kg b.wt)	-	-	**	**	***	***	***
	Glimepiride 1 mg/kg b.wt	**	**	**	**	***	***	***
**B**	**10a** (60 mg/kg b.wt)	-	-	-	-	-	-	-
	**10a** (30 mg/kg b.wt)	-	*	*	*	*	*	-
	**10b** (60 mg/kg b.wt)	-	-	-	-	-	-	-
	**10b** (30 mg/kg b.wt)	-	-	-	-	-	-	-
	**10b** (7.5 mg/kg b.wt)	-	-	-	-	-	-	-
	**10c** (60 mg/kg b.wt)	-	-	-	-	-	-	-

(**A**): * *p* < 0.05, ** *p* < 0.05 and *** *p* < 0.001 compared to control (untreated mice); (**B**): * *p*< 0.05, ** *p* < 0.05 and *** *p* < 0.001 compared to Glimepiride (standard drug).

The synthesized compounds **10a**–**c** were designed on the basis of Topliss scheme for aromatic substituent in quantitative structure activity relationship (QSAR) which considers the hydrophobicity and the electronic effect of various substituents on activity. Thus, the first analogue synthesized (compound **10b**) was the 4-chloro derivative which is more hydrophobic (positive π) and electron-withdrawing (positive σ) than hydrogen. Alternatively, the other target (compound **10c**) has a methyl substituent which is an example for a substituent with a positive π and negative σ values. Based on the *in vivo* biological evaluation of compound **10b**, it is possible to propose that hydrophobic electron withdrawing substituent would be good for activity. Therefore, future research should address optimum substituent.

The targeted compounds **10a**–**c** were designed to be tolbutamide analogues by merging the pharmacophoric features of tolbutamide with glucosamine moiety to provide higher potency and selectivity. In this study, glimepiride, the third generation sulfonylurea drug was used as a potent antihypergylcemic positive control [55]. Accordingly, the synthesized compounds were postulated to act primarily as tolbutamide by occupying sulphonylurea receptors (SUR) subunits of the ATP-sensitive potassium channel in pancreatic β-cells. Following occupation, potassium channels close and calcium channels open to enhance insulin secretion from the pancreatic β-cells. This results in exocytosis of insulin from storage granules [56]. Clearly, ATP-sensitive potassium channels are also found in cardiac, skeletal and smooth muscles. However, in these tissues the channels are composed of different SUR subunits that confer different drug sensitivities, even though, suphonylureas could have unfavourable cardiac effect [6]. Nevertheless, the presence of glucosamine moiety would assess selective uptake by β-cells and thus minimizes the adverse effect on cardiac potassium channels and so reduce its cardiotoxic side effect [56].

Based on our proposal, the synthesized compounds were expected to exhibit higher antihyperglycaemic effect in normal mice model having intact pancreatic β-cells upon comparison with diabetic mice model, where pancreatic cells were partially destroyed by streptozocine. On the contrary, *in vivo* results illustrated that the tested compounds **10a**–**c** excreted pronounced antihyperglycaemic effect in diabetic mice model upon comparison with normal mice model, in particular compound **10b**. At this stage, it is hard to hypothesize a proofed rational for the difference in the antihyperglycaemic effect observed and to suggest the exact mechanism of action of the synthesised compounds. However, we tried to postulate a plausible mechanism of action. It might be possible that the glycosylated sulfonylurea compounds exerted their hypoglcemic effect by stimulating the residual pancreatic β-cell or through an extra pancreatic mechanism, probably by exerting insulin mimetic action [57]. Previous studies on the molecular mechanism of extra pancreatic activity of some sulfonylureas suggested that these drugs could induce glucosetransporter-4 translocation from internal stores to the plasma membrane and activate the key metabolic enzymes, glycogen synthase and glycerol-3-phosphate acyltransferase [57,58]. Moreover, a study in rats had showed that glimebride stimulates glycogenesis. Furthermore, some sulphonylurea drugs were found to stimulate lipogenesis in 3T3 adipocytes [57]. Therefore, it is conceivable to propose that the hypoglycaemic effect may be attributed to an extra pancreatic mechanism in diabetic mice. This proposal could be supported by previous literature perusal, which showed that the hypoglycaemic effect of some sulfonylureas as tolbutamide is very unlikely to be via the stimulation of insulin secretion from the pancreas, but, it might be caused by an inhibition of the release of glucose from the liver. Clearly, further work should be conducted to explain the possible mechanisms of action.

## 3. Experimental Section

### 3.1. General Information

Reagent grade chemicals and solvents were purchased from Sigma-Aldrich (St. Louis, MO, USA) and used without purification. TLC was performed on silica gel F254 plates (Macherey-Nagel Inc., Bethlehem, PA, USA). Melting points were measured in open capillary tubes, using a Stuart melting point apparatus and by Differential Scanning Calorimetry (DSC). IR spectra were recorded as KBr discs on a Bruker Optik GmbH (Bruker, Ettlingen, Germany). Optical rotation was measured at ambient temperature using an AA-10 polarimeter (Optical Activity Ltd., Cambridgeshire, UK) in a cell volume of 1 mL and specific rotation are given in 10^−1^ deg·mL·g^−1^. ^1^H-NMR (300 and 500 MHz) and ^13^C-NMR (75 and 125 MHz) spectral data were recorded on Advance spectrometers (Bruker, Fallanden, Switzerland) with DMSO-*d*_6_ or D_2_O as a solvent and TMS as an internal standard. *J* values are in Hz. Elemental analyses were recorded on EuroEA 3000 elemental analyser (Milano, Italy). High resolution mass spectra (HRMS) were acquired (in positive or negative mode) using electrospray ion trap (ESI) technique by collision-induced dissociation on a Bruker APEX-4 (7-Tesla) instrument.

### 3.2. Chemistry

*2-Deoxy-2-[p-methoxybenzylidene(amino)]-d-glucopyranose* (**4**)*.* *p*-Anisaldehyde (3 mL, 23 mmol) was added to a freshly prepared aqueous solution of d-glucosaminehydrochloride (5 g, 23 mmol) dissolved in 1 M NaOH (24 mL). The mixture was stirred until crystallization began and then refrigerated overnight. Filtration, washing with cold water (20 mL) followed by EtOH:Et_2_O (1:1, 50 mL) and drying afforded compound **4** (5.26 g, 77%) as a white solid: m.p. 161–163 °C (lit. [49] m.p. 165–166 °C), IR ν_max_ 1643 cm^−^^1^ (N=C), 3317 cm^−1^ (O–H).

*1,3,4,6-Tetra-O-Acetyl-2-deoxy-2-[p-methoxybenzylidene(amino)]β-d-glucopyranose* (**5**). Compound **4** (4.0 g, 13 mmol) was added to a cooled mixture of pyridine (22 mL) and Ac_2_O (12 mL). The mixture was stirred for 1 h and then left overnight at room temperature. The yellow solution was poured into cooled water (80 mL). Filtration, washing with cold water (50 mL) and drying afforded compound **5** (5.5 g, 88%) as a white solid: m.p. 180–182 °C (lit. [49] m.p. 180–182 °C); ${\left[\alpha \right]}_{D}^{20}$ +93.0 (*c* 1.0, MeOH), lit. [49] ${\left[\alpha \right]}_{D}^{20}$ +95 (H_2_O): IR ν_max_ 1751 (C=O) cm^−1^ 1643 (C=N) cm^−1^; ^1^H-NMR (DMSO-*d*_6_. 300 MHz) δ_H_ 1.79 (3H, s, CH_3_CO), 1.99 (6H, s, CH_3_CO), 2.48 (3H, s, CH_3_CO), 3.33 (1H, m, H-2), 3.77 (3H, s, CH_3_O), 3.96 (1H, d, *J*_H-5–H-6_ *=* 11.5 Hz, H-5), 4.22 (2H, m, H-6α, H-6β), 4.91 (1H, t, *J*_H-4–H-3_ *=* 9.4 Hz, H-4), 5.38 (1H, t, *J*_H-3–H-4_ *=* 9.4 Hz, H-3), 6.04 (1H, d, *J*_H-1–H-2_ *=* 7.6 Hz, H-1), 6.95 (2H, d, *J =* 8.2 Hz, Ar-H_2_), 7.77 (2 H, d, *J =* 8.2 Hz, Ar-H_2_), 8.26 (1H, s, NCH).

*1,3,4,6-Tetra-O-acetyl-β-d-glucosamine hydrochloride* (**6**). Compound **5** (4.0 g, 8.6 mmol) was dissolved in warm acetone (36 mL) to which HCl (5 M, 2 mL) was added with the formation of a precipitate. The mixture was cooled and then Et_2_O (36 mL) was added and stirred for 2 h. Filtration, washing with Et_2_O and drying afforded compound **6** (2.9 g, 79%) as a white solid: m.p. 231–233 °C (lit. [49] m.p. 235 °C); ${\left[\alpha \right]}_{D}^{20}$ +36.6 (*c* 1.0, MeOH), lit. [49] +32° (H_2_O): IR ν_max_ 2939 cm^−1^ broad (NH_3_Cl), 1751 cm^−1^ (C=O); ^1^H-NMR (DMSO-*d*_6_. 300 MHz) δ_H_ 1.95 (6H, s, CH_3_CO), 2.00 (3H, s, CH_3_CO), 2.14 (3H, s, CH_3_CO), 3.52 (1H, t, *J*_H-2–H-3_ *=* 9.7 Hz, H-2), 3.98 (2H, m, H-6, H-5), 4.15 (1H, dd, *J*_H-6β–H-6α_ *=* 12.3 Hz, *J*_H-6–H-5_ *=* 4.1 Hz, H-6), 4.87 (1H, t, *J*_H-4–H-3_ *=* 9.8 Hz, H-4), 5.28 (1H, t, *J*_H-3–H-4_ *=* 9.8 Hz, H-3), 5.84 (1H, d, *J*_H-1–H-2_ *=* 8.8 Hz, H-1), 8.26 (3 H, s, NH_3_Cl).

#### 3.2.1. General Procedure for the Synthesis of *N*-(Arylsulfonyl)carbamoylides **8a**–**c**

Benzene sulfonamide compounds **7a**–**c** (33 mmol) and 4-(*N*,*N*-dimethylamino)pyridine (66 mmol) and diphenylcarbonate (37 mmol) in acetonitrile (40 mL) was stirred and then allowed to stand at room temperature overnight. Filtration, washing with MeOH (2 × 15 mL) and drying afforded compounds **8a**–**c**.

#### 3.2.2. Yields, Melting Points, Analytical and Spectroscopic Data of **8a**–**c**

*4-Dimethylaminopyridininum N-(benzenesulfonyl) carbamoylide* (**8a**). This compound was prepared from benzenesulfonamide **7a** (3.0 g); yield 3.5 g (60%); white solid; m.p. 214–216 °C (lit. [38] m.p. 214–217 °C); IR ν_max_ 3081 cm^−1^ (NH); 1706 cm^−1^ (C=O), 1646 cm^−1^ (amide I), 1573 cm^−1^ (amide II), 1257 cm^−1^ (S=O); ^1^H-NMR (DMSO-*d*_6_. 300 MHz) δ_H_ 3.21 (6H, s, CH_3_NCH_3_), 6.93 (2H, d, *J =* 7.6 Hz, 3,5-py), 7.3 (3H, m, Ar-H_3_), 7.8 (2H, m, Ar-H_2_), 8.7 (2H, d, *J* = 7.6 Hz, 2,6-py).

*4-Dimethylaminopyridininum N-(4-chlorophenylsulfonyl) carbamoylide* (**8b**). This compound was prepared from 4-chlorobenzenesulfonamide **7b** (3.0 g); yield 3.5 g (65%); white solid; m.p. 220–222 °C (lit. [38] m.p. 221–223 °C); IR ν_max_ 3091 cm^−1^ (NH); 1711 cm^−1^ (C=O), 1646 cm^−1^ (amide I), 1569 cm^−1^ (amide II), 1253 cm^−1^ (S=O); ^1^H-NMR (DMSO-*d*_6_. 300 MHz) δ_H_ 3.24 (6H, s, CH_3_NCH_3_), 6.97 (2H, d, *J =* 7.9 Hz, 3,5-py), 7.54 (2H, d, *J* = 8.5 Hz, 2, 6 Ar-H_2_), 7.83 (2H, d, *J* = 8.5 Hz, 3, 5 Ar-H_2_), 8.8 (2H, d, *J* = 7.9 Hz, 2,6-py).

*4-Dimethylaminopyridininum N-(4-methylphenylsulfonyl) carbamoylide* (**8c**). This compound was prepared from *p*-toluenesulfonamide **7c** (3.0 g); yield 3.9 g (68%); white solid; m.p. 216–220 °C (lit. [38] m.p. 220 °C); IR ν_max_ 3092 cm^−1^ (NH); 1694 cm^−1^ (C=O), 1645 cm^−1^ (amide I), 1575 cm^−1^ (amide II), 1260 cm^−1^ (S=O); ^1^H-NMR (DMSO-*d*_6_. 300 MHz) δ_H_ 2.5 (3H, s, CH_3_), 3.24 (6H, s, CH_3_NCH_3_), 6.97 (2H, d, *J =* 7.9 Hz, 3,5-py), 7.54 (2H, d, *J* = 8.5 Hz, 2, 6 Ar-H_2_), 7.83 (2H, d, *J* = 8.5 Hz, 3, 5 Ar-H_2_), 8.8 (2H, d, *J* = 7.9 Hz, 2,6-py).

#### 3.2.3. General Procedure for the Preparation of Arylsulfonylureates **9a**–**c**

*N*-(Arylsulfonyl)carbamoylide **8a**–**c** (5 mmol) and triethylamine (10 mmol) were added to a solution of compound **6** (6 mmol) dissolved in acetonitrile (15 mL).The mixture was refluxed for 5–30 min and then allowed to cool down. The solution was then acidified to form a precipitate. Filtration, washing and drying afforded compounds **9a**–**c**.

*1,3,4,6-Tetra-O-acetyl-2-deoxy-2-(benzenesulfonylurea)-d-*glucopyranose (**9a**). This compound was prepared from compound **6** (2.0 g) and **8a** (3.7 g); yield 2.7 g (80%); white solid; Decomposed at 210.7–215.7 °C; ${\left[\alpha \right]}_{D}^{20}$ +10.4 (*c* 1.0, MeCN); IR ν_max_ 3309 cm^−1^ (NH); 1759 cm^−1^ (C=O), 1666 cm^−1^ (amide I), 1550 cm^−1^ (amide II), 1165 cm^−1^ (S=O); ^1^H-NMR (DMSO-*d*_6_. 500 MHz) δ_H_ 1.69 (3H, s, CH_3_CO), 1.83 (3H, s, CH_3_CO), 1.92 (6H, s, CH_3_CO), 3.74 (1H, m, H-2), 3.90 (2H, m, H-5, H-6), 4.10 (1H, d, *J*_H-6–H-5_ *=* 7.6 Hz, H-6), 4.79 (1H, t, *J*_H-4–H-3_ *=* 9.7 Hz, H-4) 5.26 (1H, t, *J*_H-3–H-4_ *=* 9.7 Hz, H-3), 5.77 (1H, d, *J*_H-1–H-2_ *=* 7.9 Hz, H-1), 6.50 (1H, d, *J =* 8.5 Hz, NH), 7.58 (3H, m, Ar-H_3_), 7.83 (2H, d, *J =* 6.7 Hz, Ar-H_2_), 10.94 (1H, s, NH); ^13^C-NMR (DMSO-*d*_6_. 125 MHz) 20.80 (COCH_3_), 20.85 (COCH_3_), 20.86 (COCH_3_), 20.91 (COCH_3_), 20.99 (COCH_3_), 52.15 (C2), 61.95 (C6), 68.96 (C4), 71.75 (C5), 72.20 (C3), 91.23 (C1), 126.01 (Ar), 129.42 (Ar), 132.32 (Ar), 144.25 (CO), 169.20, 169.69, 170.00, 170.56 (COCH_3_ × 4); HMS HRMS (ESI+) *m*/*z* 553.10987 [M + Na]^+^ (C_21_H_26_N_2_NaO_12_S requires 553.11004).

*1,3,4,6-Tetra-O-acetyl-2-deoxy-2-(4-chlorophenylsulfonylurea)-d-glucopyranose* (**9b**). This compound was prepared from compound **6** (2.0 g) and **8b** (3.3 g); yield 2.6 g (80%); white solid; Decomposed at 185.3 °C; ${\left[\alpha \right]}_{D}^{20}$ +11.4 (*c* 1.0, MeCN); IR ν_max_ 3309 cm^−1^ (NH); 1759 cm^−1^ (C=O), 1666 cm^−1^ (amide I), 1550 cm^−1^ (amide II), 1165 cm^−1^ (S=O); ^1^H-NMR (DMSO-*d*_6_. 300 MHz) δ_H_ 1.69 (3H, s, CH_3_CO), 1.83 (3H, s, CH_3_CO), 1.92 (6H, s, CH_3_CO), 3.74 (1H, m, H-2), 3.90 (2H, m, H-5, H-6), 4.10 (1H, d, *J*_H-6–H-5_ *=* 7.6 Hz, H-6), 4.79 (1H, t, *J*_H-4–H-3_ *=* 9.7 Hz, H-4) 5.26 (1H, t, *J*_H-3–H-4_ *=* 9.7 Hz, H-3), 5.77 (1H, d, *J*_H-1–H-2_ *=* 7.9 Hz, H-1), 6.50 (1H, d, *J =* 8.5 Hz, NH), 7.58 (3H, m, Ar-H_3_), 7.83 (2H, d, *J =* 6.7 Hz, Ar-H_2_), 10.94 (1H, s, NH); ^13^C-NMR (DMSO-*d*_6_. 125 MHz) 20.74 (COCH_3_), 20.82 (COCH_3_), 20.88 (COCH_3_), 20.92 (COCH_3_), 20.95 (COCH_3_), 53.15 (C2), 61.93 (C6), 68.56 (C4), 71.78 (C5), 72.97 (C3), 92.23 (C1), 128.07 (Ar), 129.54 (Ar), 129.61 (Ar), 137.04 (Ar), 143.40 (C4-Ar), 141.68 (CO), 169.70, 169.75, 170.01, 170.51 (COCH_3_ × 4); Elem. Anal. for C_21_H_26_N_2_O_12_S% Cal. C, 44.65; H, 4.46; N, 4.96, Found C, 44.93; H, 4.14; N, 5.84; MS HRMS (ESI+) *m*/*z* 587.06180[M + Na]^+^ (C_21_H_25_ClN_2_NaO_12_S requires 587.07144).

*1,3,4,6-Tetra-O-acetyl-2-deoxy-2-(4-methylphenylsulfonylurea)-d-glucopyranose* (**9c**)*.* This compound was prepared from compound **6** (2.0 g) and **8b** (3.5 g); yield 2.7 g (79%); white solid; Decomposed at 217.1–221.3 °C; ${\left[\alpha \right]}_{D}^{20}$ +11.8 (*c* 1.0, MeCN); IR ν_max_ 3309 cm^−1^ (NH); 1759 cm^−1^ (C=O), 1666 cm^−1^ (amide I), 1550 cm^−1^ (amide II), 1165 cm^−1^ (S=O); ^1^H-NMR (DMSO-*d*_6_. 300 MHz): δ_H_ 1.73 (3H, s, CH_3_CO), 1.85 (3H, s, CH_3_CO), 1.96 (3H, s, CH_3_CO), 1.99(3H, s, CH_3_CO), 2.51 (3H, s, CH_3_), 3.76 (1H, m, H-2), 3.95 (2H, d, *J*_H-5–H-6_ *=* 10.8 Hz, H-5, H-6), 4.16 (1H, m, H-6), 4.88 (1H, t, *J*_H-4–H-3_ *=* 9.5 Hz, H-4), 5.30 (1H, t, *J*_H-3–H-4_ *=* 9.99 Hz, H-3), 5.82 (1H, d, *J*_H-1–H-2_ *=* 8.6 Hz, H-1), 6.50 (1H, d, *J =* 11 Hz, NH), 7.41 (2H, d, *J =* 8.1 Hz, Ar-H_2_), 7.76 (2H, d, *J =* 8.1 Hz, Ar-H_2_), 10.8 (1H, s, NH); ^13^C-NMR (DMSO-*d*_6_. 125 MHz) 20.52 (COCH_3_), 20.72 (COCH_3_), 20.83 (COCH_3_), 20.93 (COCH_3_), 21.44 (COCH_3_), 53.15 (C2), 61.95 (C6), 68.56 (C4), 71.75 (C5), 72.47 (C3), 92.23 (C1), 127.66 (C2, C6-Ar), 129.91 (C3, C5-Ar), 137.67 (C1-Ar), 144.25 (C4-Ar), 151.68 (CO), 169.20, 169.69, 170.00, 170.46 (COCH_3_ × 4); Elem. Anal. for C_22_H_28_N_2_O_12_S% Cal. C, 48.53; H, 5.18; N, 5.14, Found C, 47.59; H, 4.82; N, 4.99;MS HRMS (ESI+) *m*/*z* 543.12902 [M − H^+^]^−^ (C_22_H_28_N_2_O_12_S requires 543.12847).

#### 3.2.4. General Procedure for Deacetylationof Compounds **10a**–**c**

Compounds **9a**–**c** (0.4 mmol) in MeOH (20 mL) were added to a solution of 250 mmol NaOMe in MeOH (20 mL). The mixture was stirred and monitored by TLC (CHCl_3_–MeOH 9:1). The TLC was sprayed with a sugar detection visualizing reagent solution (thymol(0.5 g)in ethanol (95 mL) and 97% sulfuric acid (5 mL)). The TLC plate was then heated until a pink spot appeared. Neutralization with Dowex 50WX8-200 ion-exchange resin, filtration and evaporation afforded a viscous residue, which was re-dissolved in water, dried with anhydrous Na_2_SO_4_ and filtered. Purification by HPLC afforded compound **10a**–**c** as a fluffy white solid.

*2-Deoxy-2-(benzenesulfonylurea)-d-glucopyranose* (**10a**). This compound was prepared from compound **9a** (37%): Decomposed at 241.1 °C (DSC); ${\left[\alpha \right]}_{D}^{20}$ +10.7 (*c* 1.0, H_2_O): IR ν_max_ 3376 cm^−1^ (OH); 2823 cm^−1^ (NH); 1592 cm^−1^ (C=O), 1383 cm^−1^ (amide I), 1352 cm^−1^ (amide II), 1133 cm^−1^ (S=O) ^1^H-NMR (DMSO-*d*_6_. 500 MHz) δ_H_ 3.13 (1H, m, H-2), 3.51–3.64 (5H, m, H-3, H-4, H-5, H-6, H-6), 4.20 (1H, M, H-1β), 4.41 and 4.57 (1H, b, 1-OHα and β), 4.9–5.10 (2H, M, H-1α and OH), 5.48 (1H, b, OH), 5.87 (1H, b, OH), 6.31 (0.6H, b, NH), 7.37 (3H, m, Ar-H_3_), 7.51 (0.4H, b, NH), 7.73 (2H, m, Ar-H_2_), 8.5 (1H, s, NHC=O); ^13^C-NMR (DMSO-*d*_6_. 125 MHz) 61.69 (C6), 70.91, 71.71, 72.39, 72.78, 77.08, 83.79 (C1_β_), 91.75 (C1_α_), 126.78 (Ar), 126.88 (Ar), 128.00 (Ar), 128.05 (Ar),129.69 (Ar), 166.54 (CO); MS HRMS (ESI+) *m*/*z* 385.06761 [M + Na]^+^ (C_13_H_18_N_2_NaO_8_S requires 385.06815).

*2-Deoxy-2-(4-chlorophenylsulfonylurea)-d-glucopyranose* (**10b**)*.* This compound was prepared from compound **9b** (71%): Decomposed at 189.4 °C (DSC); ${\left[\alpha \right]}_{D}^{20}$ +10.95 (*c* 1.0, H_2_O): IR ν_max_ 3421 cm^−1^ (OH); 2831 cm^−1^ (NH); 1601 cm^−1^ (C=O), 13361 cm^−1^ (amide I), 1245cm^−1^ (amide II), 1135 cm^−1^ (S=O); ^1^H-NMR (DMSO-*d*_6_. 500 MHz) δ_H_ 3.10 (1H, m, H-2), 3.40–3.70 (5H, m, H-3, H-4, H-5, H-6, H-6), 4.20 (1H, M, H-1β), 4.42 and 4.45 (1H, b, 1-OHα and β), 4.9–5.10 (2H, M, H-1α and OH), 5.40 (1H, b, OH), 5.89 (1H, b, OH), 6.41 (1H, b, NH), 7.42 (2H, d, *J =* 8.4 Hz, Ar-H_2_), 7.8 (2H, d, *J =* 8.4 Hz, Ar-H_2_), 10.8 (1H, s, NHC=O); ^13^C-NMR (DMSO-*d*_6_. 125 MHz) 61.69 (C6), 70.92 (C2), 71.66 (C5), 72.41 (C4), 77.06 (C3), 91.74 (C1α), 95.74 (C1β), 128.02 (Ar), 128.96 (Ar), 134.31 (Ar), 166.83 (CO); MS HRMS (ESI+): *m*/*z* 419.02864 [M + Na]^+^ (C_13_H_17_ClN_2_NaO_8_S requires 419.02918).

*2-Deoxy-2-(4-methylphenylsulfonylurea)-d-glucopyranose* (**10c**). This compound was prepared from compound **9c** (81%): Decomposed at 230.9 °C (DSC); ${\left[\alpha \right]}_{D}^{20}$ +11.2 (*c* 1.0, H_2_O): IR ν_max_ 3421 cm^−1^ (OH); 2831 cm^−1^ (NH); 1601 cm^−1^ (C=O), 13361 cm^−1^ (amide I), 1245cm^−1^ (amide II), 1135 cm^−1^ (S=O); ^1^H-NMR (DMSO-*d*_6_. 500 MHz) δ_H_ 2.51 (3H, s, CH_3_), 2.54(1H, m, H-2), 2.72–3.23 (5H, m, H-3, H-4, H-5, H-6, H-6), 4.20 (1H, M, H-1β), 4.42 and 4.87 (1H, b, 1-OHα and β), 4.9–5.12 (2 H, M, H-1α and OH), 5.40 (1H, b, OH), 5.9 (1H, b, OH), 6.41 (1H, b, NH), 7.15 (2H, m, Hz, Ar-H_2_), 7.59 (2H, d, *J =* 7.8 Hz, Ar-H_2_), 8.49 (1H, s, NH), ^13^C-NMR (DMSO-*d*_6_. 125 MHz) 23.28 (COCH_3_), 60.61 (C6), 69.94 (C2), 70.05 (C5), 71.54(C4), 75.84(C3), 91.53 (C1_β_), 93.74 (C1_α_), 126.08 (Ar), 129.36 (Ar), 142.87 (Ar), 142.90 (Ar), 171.07 (CO); MS HRMS (ESI+): *m*/*z* 399.08326 [M + Na]^+^ (C_14_H_20_ClN_2_NaO_8_S requires 399.08380).

### 3.3. In Vivo Testing

#### 3.3.1. Chemicals

Streptozotocin (STZ) was purchased from Sigma-Aldrich, Glimperide was kindly donated by Dr Ismail M. Khalifeh from Dar Al Dawa Development & Investment Company^®^ (Amman, Jordan).

#### 3.3.2. Animals

A total of 90 Balb/cmale mice, weight between 20–30 g were used in all experiments. The animals were purchased from the Animal House in Applied Science University. The *in vivo* testing was conducted in the animal house of the Faculty of Medicine at The University of Jordan. All animals were acclimatized for a week before use and were maintained in hygienic conditions at room temperature, fed with standard pellets and tap water in accordance with the in-house ethical guidelines for animal protection. The study was conducted after obtaining Institutional animal ethical committee’s clearance by the Scientific Research Committee at the Faculty of Pharmacy, The University of Jordan. Animals were deprived from food and water for 18 h before *in vivo* initial glycemia determination. Blood glucose level from cut tail tips was determined using an Accu-Chek^®^ Active Glucose meter.

#### 3.3.3. Oral Glucose Tolerance Test

Experimental mice were divided into two groups. Group A contained normal mice and group B contained STZ-induced diabetic mice. In each group, mice were randomly divided into five cages I-V (*n* = 6 mice per subgroup). Experimental mice were fasted overnight (18 h) and then, initial glycemia was determined (0 min). After which, glimperide (standard antihyperglycaemic drug, 1 mg/kg b.wt.) and compounds **10a**–**c** at different doses 60, 30 and 7.5 mg/kg b.wt. were dissolved in water for injection and directly administered intraperitoneally. Control untreated mice received the vehicle (water). At 30 min time point of the test, glucose (2 g/kg b.wt.) was administered orally via intra-gastric intubation to all test mice groups [54].

#### 3.3.4. Induction of Diabetes in Mice

Diabetes in group B mice was induced by single intraperitoneal (IP) injection of freshly prepared STZ (200 mg/kg b.wt) in 200 µL 0.1 M citrate buffer pH 4.5. Mice were supplied orally with glucose solution (2 g/kg) for 48 h after STZ injection in order to prevent hypoglycemia. After 7 days, blood glucose level from cut tail tips was measured followed by daily measurement until autopsy. Mice with permanent fasting blood glucose level (FBGL) above 280 mg/dL were considered as diabetic and included in this study. Negative control mice were treated with the vehicle only [55,59].

#### 3.3.5. Blood Collection and Determination of Blood Glucose

Blood glucose level from cut tail tips was monitored using an Accu-Chek^®^ Active glucometer [59]. The percentage (%) change of glucose level from the initial glycaemia was calculated using the following formula: % glycaemia change = (G_x_− G_o_)/G_o_ × 100. G_o_ is the initial glycaemia value at zero time after overnight fasting; G_x_ is the glycaemia value at x minutes after vehicle or tested compounds administration [1,60,61].

#### 3.3.6. Statistical Analysis

Experimental results were expressed as mean ± SEM. The data were analyzed by ANOVA (*p* < 0.05) and means separated by Dunnett multiple range tests (by SPSS version 16 software, SPSS Inc., Chicago, IL, USA).

### 3.4. High Performance Liquid Chromatography (HPLC)

An integrated HPLC system (Thermo Fisher Scientific, Waltham, MA, USA) equipped with a Surveyor LC pump, Surveyor auto sampler, UV 6000 LP and Surveyor UV-VIS photodiode array detector was used with a Hypersil^®^ reverse phase C18 column (250 mm × 4.6 mm, 5 μm). Mobile phase was prepared with buffer, ACN, THF (40:50:10). Buffer was prepared by dissolving 7.1 g of K_2_HPO_4_ in 1 L of water and the pH was adjusted to 5.0 with H_3_PO_4_. The mobile phase was filtered through a 0.45 µm filter (Supelco, Bellefonte, PA, USA).The flow rate was 1.0 mL/min. The injection volume was 20 µL. Absorbance was monitored at 254 nm at 25 °C [62]. Semi preparative RP-HPLC was conducted on a Waters SymmetryPrep™ C8, 7 μm, 19 × 300 mm column using water/ACN gradients at a flow rate of 7 mL/min.

## 4. Conclusions

In the present study, we have synthesized and evaluated novel class of glycosylated aryl sulfonylurea antidiabetic agents. Compounds **10a**–**c** exhibited antihyperglycemic activity in streptozotocin-induced diabetic mice. The percentage blood glucose reduction induced by the tested compounds in streptozotocin-induced diabetic mice is greater than that observed in normal treated mice. Although, assay of the changes in blood glucose level was a regular method for detecting the hypoglycaemic effect of the tested compounds, there is still a need for future work such as the assay of blood insulin level which could provide us with a more reliable explanation for the observed activity. The highest antihyperglycemic activity was achieved by compound **10b**. This investigation thus indicates the importance of these novel compounds as potential lead candidates.
